# Depression in multiple sclerosis: RS-FMRI research

**DOI:** 10.1192/j.eurpsy.2021.265

**Published:** 2021-08-13

**Authors:** A. Temniy, K. Markin, M. Poplyak, A. Trufanov, D. Tarumov, I. Litvinenko

**Affiliations:** 1 Psychiatry, S.M.Kirov Military Medical Academy, Saint-Petersburg, Russian Federation; 2 Neurology, S.M.Kirov Military Medical Academy, Saint-Petersburg, Russian Federation

**Keywords:** Multiple sclerosis, Depression, rs-fMRI, comorbidity

## Abstract

**Introduction:**

Multiple sclerosis (MS) is a demyelinating and neurodegenerative disorder of the CNS, which incapacitates people of working age. Due to progressive disability, the quality of life decreases, adding a number of other diseases to the main one. Several studies have reported high rates of depression in MS with a lifetime prevalence of approximately 50%.

**Objectives:**

Therefore, we would like to pattern the functional activation of the brain of patients with different phenotypes of MS. This would objectify the patient’s condition and the effectiveness of therapy for these diseases.

**Methods:**

68 patients with MS were examined: 40 with a relapsing-remitting type of course (RRMS) in remission and 28 with secondary - progressive MS (SPMS). Patients underwent MRI of the brain on a Siemens Tim Trio 3.0 T tomograph and processed the data using CONN 18b software. Clinical features were estimated by tests (BDI, HADS) results.

**Results:**

91% of all MS patients in research have signs of depression. We noted that decreased FC in RRMS patients has a whole-brain type, but it is only decreasing, not losing the connections between brain clusters. Decreased FC and losing the connections between large-scale brain networks and brain clusters. Due to tests, more severe depression was observed in SPMS patients.

**Conclusions:**

Our findings suggest that patients with SPMS have depression, cause of decreasing in FC between the main clusters of the brain, and patients with SPMS have more severe depression, which, as we assume, neurodegeneration has turned into atrophy and loosing all connections between clusters even in large-scale brain networks.

**Disclosure:**

No significant relationships.

